# Solar Panel Technologies for Light-to-Chemical Conversion

**DOI:** 10.1021/acs.accounts.2c00477

**Published:** 2022-11-17

**Authors:** Virgil Andrei, Qian Wang, Taylor Uekert, Subhajit Bhattacharjee, Erwin Reisner

**Affiliations:** Yusuf Hamied Department of Chemistry, University of Cambridge, Lensfield Road, CambridgeCB2 1EW, United Kingdom

## Abstract

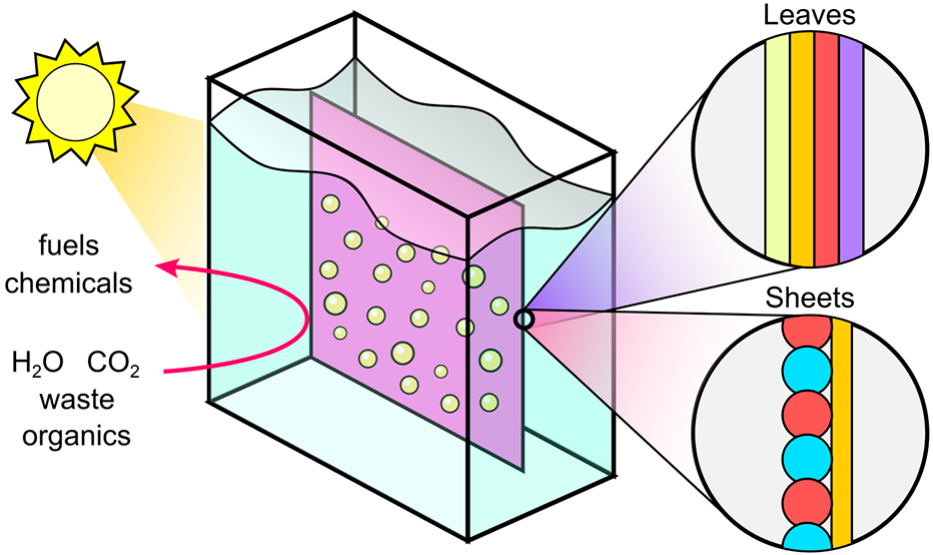

The sustainable synthesis of fuels and chemicals is key to attaining a carbon-neutral economy. This can be achieved by mimicking the light-harvesting and catalytic processes occurring in plants. Solar fuel production is commonly performed via established approaches, including photovoltaic–electrochemical (PV–EC), photoelectrochemical (PEC), and photocatalytic (PC) systems. A recent shift saw these systems evolve into integrated, compact panels, which suit practical applications through their simplicity, scalability, and ease of operation. This advance has resulted in a suite of apparently similar technologies, including the so-called artificial leaves and PC sheets. In this Account, we compare these different thin film technologies based on their micro- and nanostructure (i.e., layered vs particulate), operation principle (products occurring on the same or different sides of the panel), and product/reaction scope (overall water splitting and CO_2_ reduction, or organics, biomass, and waste conversion).

For this purpose, we give an overview of developments established over the past few years in our laboratory. Two light absorbers are generally required to overcome the thermodynamic challenges of coupling water oxidation to proton or CO_2_ reduction with good efficiency. Hence, tandem artificial leaves combine a lead halide perovskite photocathode with a BiVO_4_ photoanode to generate syngas (a mixture of H_2_ and CO), whereas PC sheets involve metal-ion-doped SrTiO_3_ and BiVO_4_ particles for selective formate synthesis from CO_2_ and water. On the other hand, only a single light absorber is needed for coupling H_2_ evolution to organics oxidation in the thermodynamically less demanding photoreforming process. This can be performed by immobilized carbon nitride (CN_*x*_) in the case of PC sheets or by a single perovskite light absorber in the case of PEC reforming leaves. Such systems can be integrated with a range of inorganic, molecular, and biological catalysts, including metal alloys, molecular cobalt complexes, enzymes, and bacteria, with low overpotentials and high catalytic activities toward selective product formation.

This wide reaction scope introduces new challenges toward quantifying and comparing the performance of different systems. To this end, we propose new metrics to evaluate the performance of solar fuel panels based on the areal product rates and commercial product value. We further explore the key opportunities and challenges facing the commercialization of thin film technologies for solar fuels research, including performance losses over larger areas and catalyst/device recyclability. Finally, we identify emerging applications beyond fuels, where such light-driven panels can make a difference, including the waste management, chemical synthesis, and pharmaceutical industries. In the long term, these aspects may facilitate a transition toward a light-driven circular economy.

## Key References

- AndreiV.; ReuillardB.; ReisnerE.Bias-Free Solar Syngas Production by Integrating a Molecular Cobalt Catalyst with Perovskite–BiVO_4_ Tandems. Nat. Mater.2020, 19, 189–194.3163642310.1038/s41563-019-0501-6(1)*Demonstration of an unassisted perovskite–BiVO_4_ PEC artificial leaf. An integrated perovskite photocathode enables the device to couple O_2_ evolution to the challenging aqueous CO_2_ reduction.*
- WangQ.; WarnanJ.; Rodríguez-JiménezS.; LeungJ. J.; KalathilS.; AndreiV.; DomenK.; ReisnerE.Molecularly Engineered Photocatalyst Sheet for Scalable Solar Formate Production from Carbon Dioxide and Water. Nat. Energy2020, 5 ( (9), ), 703–710.^2^*A molecular cobalt catalyst integrated on a wireless, monolithic photocatalyst sheet based on SrTiO_3_:La,Rh and BiVO_4_:Mo particles, enabling selective solar-driven CO_2_-to-formate conversion coupled to water oxidation.*
- UekertT.; BajadaM. A.; SchubertT.; PichlerC. M.; ReisnerE.Scalable Photocatalyst Panels for Photoreforming of Plastic, Biomass and Mixed Waste in Flow. ChemSusChem2021, 14 ( (19), ), 4190–41973315656210.1002/cssc.202002580.^3^*Immobilized CN_x_ panels produce H_2_ and oxidize waste under flow during solar irradiation, overcoming the recyclability challenge of particulate systems.*
- BhattacharjeeS.; AndreiV.; PornrungrojC.; RahamanM.; PichlerC. M.; ReisnerE.Reforming of Soluble Biomass and Plastic Derived Waste Using a Bias-Free Cu_30_Pd_70_|Perovskite|Pt Photoelectrochemical Device. Adv. Funct. Mater.2022, 32 ( (7), ), 2109313.^4^*A PEC leaf employing a single perovskite light absorber demonstrates selective biomass and PET reforming while producing H_2_ at rates higher than for conventional PC approaches.*

## 1 Introduction

While renewable electricity is becoming more widespread, aviation, shipping, and the chemical industries still rely heavily on conventional fuels. Hence, solar-driven chemical synthesis will become a crucial contributor to attaining a circular economy. Solar fuels research has been pursued ever since the initial studies on solar water splitting with TiO_2_ photoelectrodes by Fujishima and Honda 50 years ago.^5^ Since then, PV–EC, PEC, and PC systems stood out as the most common approaches for solar-to-chemical conversion.^6^ However, overall fuel production limits the choice of single light absorbers to wide bandgap semiconductors absorbing mainly in the UV range.^5,7^ Furthermore, many studies focused only on photoelectrodes and photocatalyst powders with sacrificial reagents for either proton/CO_2_ reduction or water oxidation.^8^

Complementary light absorbers can be combined to attain a higher coverage of the visible spectrum, with early multijunction systems already reaching 18.3% efficiency for overall water splitting in 2000.^9^ These light harvesters can also be interfaced directly or indirectly to biological systems such as bacteria, producing organics at efficiencies beyond 4%, which exceeds that of natural photosynthesis.^10,11^ However, this approach requires a more complex wiring and reactor design in the case of PV–EC systems and photoelectrodes^12^ as well as a careful choice of redox mediators for PC suspensions,^13,14^ thereby limiting their implementation to laboratory prototypes.

**Figure 1:**
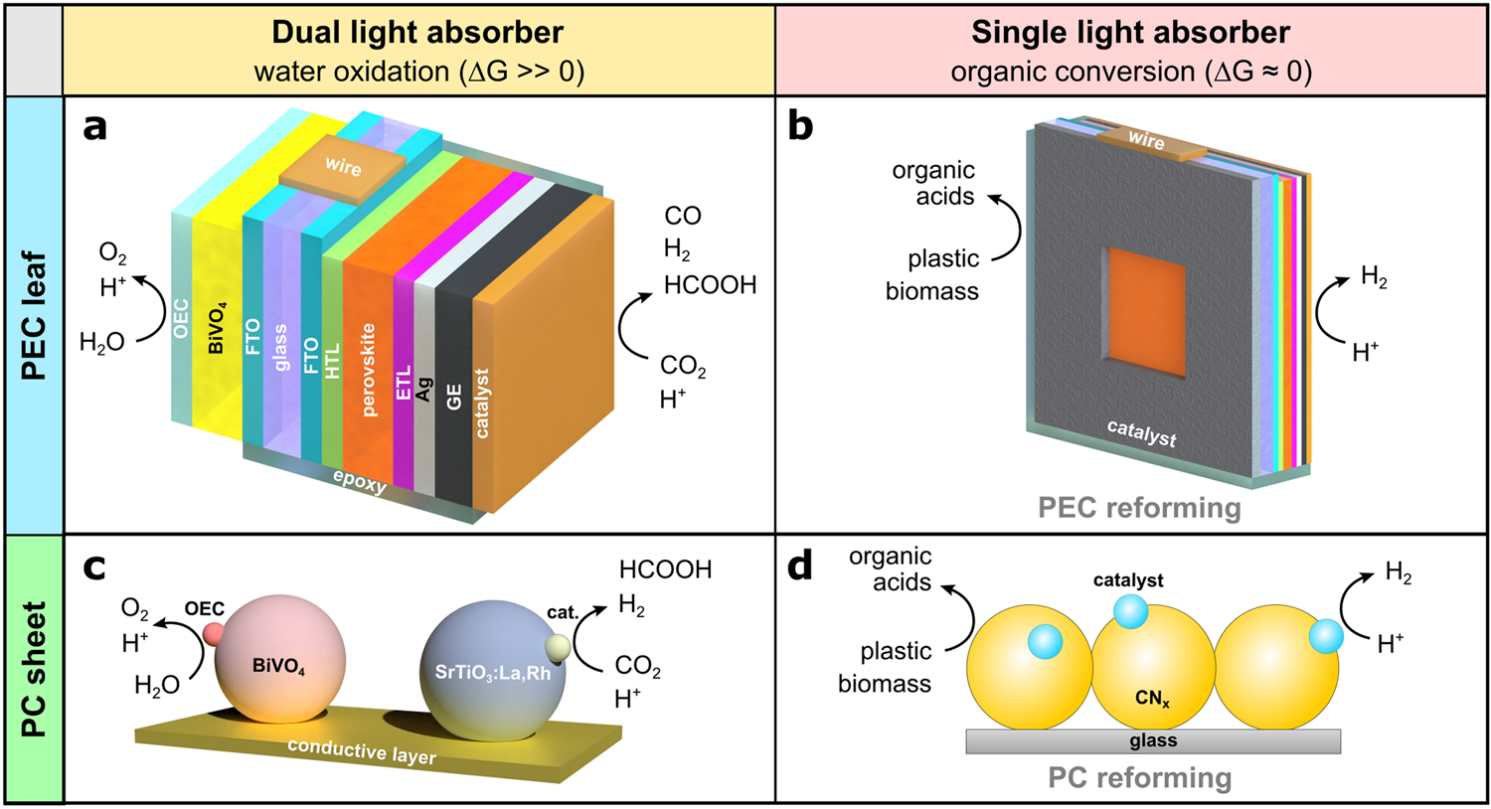
Examples of standalone thin film technologies described in this Account: (a, b) PEC artificial leaves, (c, d) PC sheets. (a) A tandem BiVO_4_–perovskite device can produce O_2_ and syngas (H_2_ + CO) or formate from water and CO_2_. (b) A single perovskite light absorber simultaneously performs proton reduction and organics oxidation. The perovskite PV device structure corresponds to the one in (a), but the BiVO_4_ photoanode is replaced by a metal alloy electrocatalyst. (c) Doped BiVO_4_ and SrTiO_3_ powders are interfaced through a solid gold layer, achieving hydrogen or selective formate production. (d) Immobilized semiconductor particles combine H_2_ evolution with the reforming of organics. Abbreviations: OEC, oxygen evolution catalyst; FTO, fluorine-doped tin oxide; HTL/ETL, hole/electron transport layer; GE, graphite epoxy paste (conductive encapsulant); and CN_*x*_, carbon nitride.

Over the past decade, these three distinct technologies began undergoing the next stage of development, evolving toward integrated, compact systems that are more suitable for wider applications. By removing wiring, thin film light absorbers could be interfaced directly with suitable electrocatalysts to form the well-known artificial leaves, a term introduced in 2011.^15,16^ On the other hand, practical PC water splitting could be demonstrated by immobilizing semiconductor particles onto a thin solid conductive film (Figure 1), as reported in 2016.^17,18^

In this Account, we distill the common design principles of such thin film technologies including artificial leaves consisting of multiple thin layers of materials and photocatalyst sheets containing a layer of semiconductor particles, assessing their individual prospects and challenges for solar fuel production and proposing alternative performance metrics. On this occasion, we summarize our recent progress in expanding the scope of these technologies beyond H_2_ production and discuss solar chemical applications more broadly. In addition, we showcase oxidative organic transformations including biomass^19,20^ or plastic^21^ photoreforming as a way to circumvent the thermodynamic limitations imposed by water oxidation and explore how this can be expanded to flat panel systems.

## 2 Thin Film Technologies

Solar fuels can be produced using a wide range of configurations, which makes an unequivocal classification of those systems challenging. While several nomenclatures have already been reported for solar fuel systems,^22,23^ we propose a simple classification based on the oxidation reaction and device structure. The distinct thermodynamic requirements of organics oxidation and O_2_ evolution may demand a single or tandem light absorber configuration, whereas the starting material could be more suitable for a layered or particulate panel.

### 2.1 Water Oxidation Coupled to H^+^/CO_2_ Reduction

#### 2.1.1 Artificial Leaves

The artificial leaf design was experimentally demonstrated by sandwiching a triple-junction amorphous silicon solar cell between NiMoZn and Co-based catalysts.^15,16^ Ever since, a broad community has started developing integrated devices,^24^ expanding this concept to a wide range of light absorbers in PEC or PV–PEC configurations. Despite solar-to-hydrogen conversion efficiencies reaching beyond 3% for water splitting (Table 1),^25^ CO_2_ reduction posed challenges for artificial leaf devices using only two light absorbers. This occurred because most conventional narrow bandgap light absorbers (such as Si, Cu_2_O, or dyes) provide photovoltages below 0.7 V,^26^ whereas an additional overpotential of ∼0.4 V must be overcome for commonly employed CO production catalysts. Instead, several studies focused on CO_2_ reduction to formate, which could be accomplished at low overpotentials using tandem SrTiO_3_–InP^27^ and triple-junction amorphous SiGe^28^ leaves.

**Table 1:** Performance Metrics for Some Representative Solar Fuel Systems (PV Estimate Given for Comparison)^a^^,^^b^

sample	η_STF_	product	product rate	value rate	comments	ref
	(%)		(μmol cm^–2^ h^–1^)	($ cm^–2^ h^–1^)		
**Artificial Leaves**						
Co|3jn-a-Si|NiMoZn	2.5	H_2_, O_2_			0.5 M KB_i_, 1.5 M KNO_3_	(15)
IrO_*x*_|3jn-a-SiGe|CC|RuCP	4.6	HCOOH (O_2_)	59.7	1.32 × 10^–6^	CO_2_ sat. 0.1 M KP_i_, pH 6.4	(28)
Mo:BiVO_4_-PVK-Pt	3	H_2_	41.9	2.57 × 10^–7^	0.1 M KHCO_3_, pH 7	(25)
		O_2_	22.0			
BiVO_4_-PVK|FM|CoMTPP@CNT	0.018	CO	0.18	5.14 × 10^–9^	0.5 M KHCO_3_, pH 7.4	(1)
	0.056	H_2_	0.58			
	0.146	O_2_	0.68			
BiVO_4_-PVK|GE|Pt	1.26	H_2_	10.2	6.20 × 10^–8^	0.1 M KB_i_, 0.1 M K_2_SO_4_, pH 8.5	(31)
		O_2_	4.95			
BiVO_4_-PVK|GE|IO-TiO_2_|FDH	0.80	HCOOH (O_2_)	7.1	1.57 × 10^–7^	MOPS, NaHCO_3_, CsCl, pH 6.4	(33)
Pt|PVK|Cu_30_Pd_70_	n.a.	H_2_	43.8	6.32 × 10^–6^	cellulose, 1 M KOH	(4)
		gluconic acid	26.8			
Pt|PVK|Cu_30_Pd_70_	n.a.	H_2_	70.5	2.52 × 10^–7^	PET bottle, 1 M KOH	(4)
		glycolic acid	30.5			
**PC Sheets**						
RhCrO_*x*_|CoO_*y*_|SrTiO_3_:Al	0.4	H_2_ (O_2_)	4.2	2.53 × 10^–8^	∼0.7 sun (outdoors), H_2_O	(36)
Cr_2_O_3_|Ru-SrTiO_3_:La,Rh|Au|BiVO_4_:Mo	1.1	H_2_	19.6	1.19 × 10^–7^	H_2_O, 331 K, 10 kPa	(17)
		O_2_	9.8			
CotpyP-SrTiO_3_:La,Rh|Au|BiVO_4_:Mo	0.08	HCOOH	1.09	3.26 × 10^–8^	0.1 M KHCO_3_, pH 6.7	(2)
		H_2_	0.03			
		O_2_	0.52			
CN_*x*_|Ni_2_P panel	n.a.	H_2_	0.02	2.20 × 10^–9^	PET, 0.5 M KOH	(3)
		HCOOH (mixture)	0.09			
**PV Panels**		electricity		1.09 × 10^–6^	assuming $0.0685 kWh^–1^ cost	

a Value of chemicals ($ kg^–1^), estimated based on NREL procedures:^56^ CO_2_, 0.17; CO, 0.44; H_2_, 2.52; O_2_, 0.06; H_2_O, 2.9 × 10^–4^; formic acid, 0.63; cellulose, 0.90; PET, 0.27; glycolic acid, 0.63; gluconic acid, 1.99.

b Abbreviations: jn, junction; a, amorphous; KB_i_, potassium borate buffer; CC, carbon cloth; RuCP, Ru complex polymer; PVK, perovskite; and n.a., not applicable.

Our laboratory could overcome these challenges by introducing lead-halide perovskite photocathodes, which were combined with BiVO_4_ photoanodes in back-to-back, two-electrode tandem PEC devices (Figure 1a).^1,29^ In this arrangement, a buried-PV electrode was constructed by encapsulating a perovskite PV cell with a conductive layer (either Field’s metal^1,29^ or graphite epoxy paste^30,31^), which provided an interface to molecular and inorganic electrocatalysts with low overpotentials toward CO_2_ reduction. The thin graphite paste encapsulation enabled a redesign of the device structure, resulting in lightweight artificial leaves which stand out in terms of cost, scalability, and functionality.^32^ This arrangement could be expanded to semi-artificial photosynthesis systems interfacing semiconductors to enzymes with very low overpotentials for proton or CO_2_ reduction.^33−35^ By integrating hydrogenase in a hierarchically structured inverse opal TiO_2_ scaffold, such systems could perform water splitting at 1.1% solar-to-hydrogen efficiency,^34^ whereas a 0.8% solar-to-formate efficiency and a Faradaic yield of 83% were attained with formate dehydrogenase.^33^

#### 2.1.2 PC Sheets

Particulate photocatalysts can also be used to produce solar fuels from water and CO_2_. In such processes, the reduction and oxidation reactions typically take place in close proximity to one another using redox mediators, preventing concentration overpotentials and avoiding the use of an electrolyte.^7^ To ensure adequate mass transfer, photocatalytic processes are typically carried out by dispersing the photocatalyst powder in a reaction solution. While a powder suspension is the simplest way to generate solar fuels, it has several disadvantages in terms of recycling and large-scale applications. One strategy for tackling these issues is to fabricate a photocatalyst sheet by fixing the photocatalyst powder on a substrate.^36,37^ A recent milestone demonstrated a 100 m^2^ array of Al-doped SrTiO_3_ photocatalyst sheets that generated solar H_2_ over several months via water splitting, showing a maximum solar-to-hydrogen conversion efficiency of 0.76%.^38^

Additionally, photocatalytic processes can be accomplished by employing a nature-inspired Z-scheme configuration, consisting of two distinct photocatalysts connected by a redox mediator as interparticle electron relay. Z-scheme photocatalyst sheets (Figure 1c) comprise two photocatalysts embedded in a conductive layer, such as gold or carbon, to ensure interparticle electron transfer while avoiding the side reactions caused by redox mediators.^17,18^ One example is a photocatalyst sheet containing SrTiO_3_:La,Rh and BiVO_4_:Mo that is fixed on a Au layer and achieves a solar-to-hydrogen conversion efficiency of more than 1%.^17^

Light-driven fuel production from CO_2_ is currently hampered by sacrificial electron donor utilization and low efficiency, selectivity, and scalability. To overcome these barriers, our laboratory integrated a selective molecular catalyst (phosphonated cobalt(II) bis(terpyridine), CotpyP) on semiconductor light absorbers to form a wireless, monolithic photocatalyst sheet (CotpyP-SrTiO_3_:La,Rh|Au|BiVO_4_:Mo).^2^ The device combines the high selectivity of molecular catalysts for CO_2_ reduction and the strong water oxidation ability of semiconductors, resulting in a solar-to-formate conversion efficiency of 0.08 ± 0.01% with a selectivity for formate of 97 ± 3%. Furthermore, we developed an approach to produce multicarbon products by combining photocatalyst sheets (SrTiO_3_:La,Rh|ITO|BiVO_4_:Mo) capable of photocatalytic water splitting with the non-photosynthetic, CO_2_-fixing acetogenic bacterium *Sporomusa ovata* as a living biocatalyst. The resulting semi-biological hybrid system combines the light-harvesting ability of the photocatalyst sheet with the high selectivity of the biological catalyst, thereby achieving a solar-to-acetate conversion efficiency of ∼0.7% with high selectivity for acetate production.^39^

### 2.2 Organics Oxidation Coupled to H^+^/CO_2_ Reduction

Coupling water or CO_2_ reduction with the oxidation of organic substrates can lower the required cell potential to near-neutral levels while also promoting the formation of useful organic products. Waste streams such as plastics, biomass, food, and mixed municipal solids are particularly desirable substrates as they contain oxidizable organic molecules of the form C_*x*_H_*y*_O_*z*_, are freely available, and require creative mitigation solutions.^40^

#### 2.2.1 Artificial Leaves

There have been a few reports on two-compartment PEC systems for H_2_ evolution, which utilize a simple organic substrate such as glucose^41−43^ or glycerol^44−46^ instead of water oxidation. However, most of those PEC systems require an additional external energy input in the form of an applied bias voltage,^41,43−45^ employ a tandem light absorber configuration,^42,47^ or exhibit low efficiencies and product selectivities.^41−43,46,47^ Furthermore, a two-compartment arrangement may be impractical for large-scale applications.

**Figure 2:**
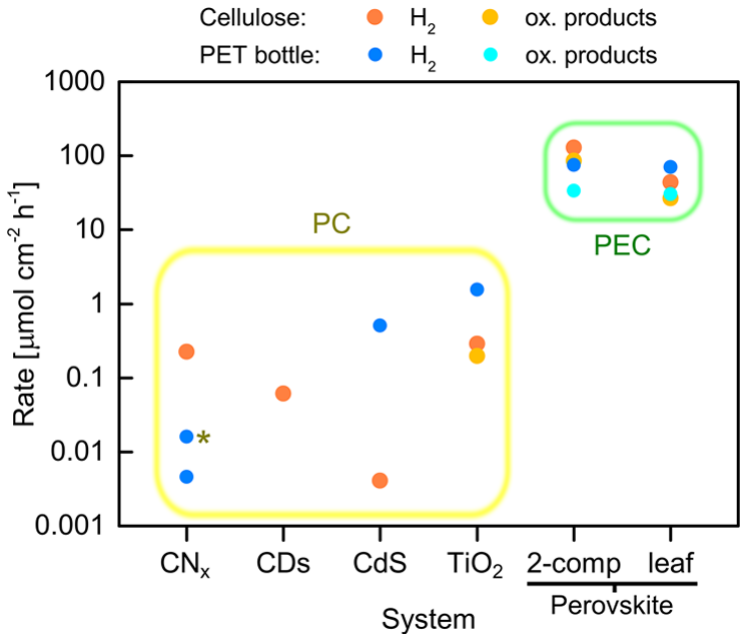
Comparison between the product rates of suspension and thin film technologies for photoreforming. Waste conversion can be performed with semiconductor particles (that is, PC reforming) or with integrated artificial leaves (PEC reforming). The star indicates a PC sheet. CDs, carbon dots; PET, poly(ethylene terephthalate). Adapted with permission from ref (4). Copyright 2022 the Authors. Published by Wiley under a Creative Commons Attribution 4.0 International (CC BY 4.0) License.

We have recently developed a single-light-absorber PEC device that can reform a diverse range of pretreated waste substrates and simultaneously generate H_2_. These Cu_30_Pd_70_|perovskite|Pt leaves integrate a perovskite photocathode with a Cu_30_Pd_70_ anode (Figure 1b). The devices can convert glycerol (a by-product from the biofuel industry), poly(ethylene terephthalate) (PET) bottles, and cellulose (a component of lignocellulosic biomass) to value-added products such as glyceric acid, glycolic acid, and gluconic acid, with a product selectivity between 60 and 90%.^4^ These bias-free PEC systems achieve 10^2^–10^4^ times higher product formation rates over those of conventional waste photoreforming approaches based on particulate photocatalysts (Figure 2). Another important aspect of this system is its versatility in terms of assembly. The PEC device can be constructed in either a two-compartment configuration or as a standalone artificial leaf. The former is suitable for non-transparent waste streams, whereas the latter allows for facile device retrieval and reuse.

#### 2.2.2 PC Sheets

As with PEC systems, the application of photocatalyst sheets for simultaneous H_2_ production and organics oxidation remains limited. This can be attributed to several challenges, including reduced mass transfer between the catalyst and reactant, parasitic light absorption by the reactant, and practical deposition difficulties. Nevertheless, a variety of TiO_2_ sheets have been applied to the photodegradation of organic pollutants.^48^ H_2_ generation with a triethanolamine electron donor was also reported with PC sheets prepared by drop-casting mesoporous CN_*x*_ coupled with a Pt co-catalyst onto stainless steel with a Nafion binder.^49^ Recently, we have utilized a similar drop-casting technique to prepare CN_*x*_|Ni_2_P sheets of up to 5 × 5 cm^2^ (Figure 1d). By illuminating these PC sheets from behind, this system was able to successfully photoreform turbid PET, biomass, and mixed municipal waste streams into H_2_ and various small organics such as formate, carbonate, and glyoxal.^3^ CN_*x*_|Pt and TiO_2_|Pt sheets prepared by the same strategy were also shown to produce H_2_, ethane, and ethylene from polyethylene-derived organic acids.^50^

## 3 Assembly and Characterization

### 3.1 Assembly

As a key common feature, these thin film technologies take the shape of a compact panel, where the precise assembly of the light-harvesting and catalytic components governs the reactivity. To prepare an artificial leaf, the individual components of an unassisted PV–EC or PEC system must be integrated in a standalone device. Such devices can be fabricated in a wireless, monolithic design, where the catalysts are interfaced to both sides of the light harvester.^15,28^ The same functionality can be obtained by attaching a photocathode to a suitable (photo)anode in a back-to-back, tandem device configuration.^29^ In this case, the front electrode (typically a wide bandgap photoanode) needs to be partially transparent, while the connection between both electrodes is made through a short, embedded wire.^1^ Those individual photoelectrodes can be fabricated by conventional deposition techniques including spin coating, electrodeposition, drop casting, and thermal evaporation.^29,51^

Transitioning from powder suspensions to PC sheets requires immobilization of the particles onto a substrate. This may require further post-annealing treatment, binders, and textured substrates such as frosted glass^3,38^ to increase the photoactive area and improve semiconductor adhesion. If two different light absorber particles are used, then a conductive interface is also necessary. This can be provided by the thermal evaporation of a Au^2,17^ or carbon layer,^18^ or the addition of indium tin oxide (ITO)^52^ nanoparticles. In the case of overall water splitting, care must be taken to prevent the backward reaction (O_2_ reduction and H_2_ oxidation), as H_2_ and O_2_ are produced in close proximity. This can be prevented by the photodeposition of Cr_2_O_3_ and TiO_2_ surface modifiers. The hydrated oxide layers hinder O_2_ penetration while allowing protons to reach the catalytic centers for H_2_ production.^17^

### 3.2 Characterization and Product Quantification

Prior to the assembly of bias-free, two-electrode PEC systems or standalone artificial leaves, the respective cathodic and anodic processes should be thoroughly analyzed to determine the exact operating conditions (working potentials and expected current densities) during unassisted operation. For this purpose, cyclic voltammetry (CV) scans of the individual (photo)electrodes must be taken in a two-compartment, three-electrode setup, where Ag/AgCl and Pt can act as reference and counter electrodes, respectively. The overlap between the individual CV scans of the (photo)anode and (photo)cathode corresponds to the ideal current density during bias-free operation of the artificial leaf. This overlap also determines the potential applied to the catalysts during operation; hence, the product selectivity can be steered by adjusting the photovoltage and catalyst overpotential. For wired PEC devices, chronoamperometry is performed under simulated sunlight irradiation (often AM 1.5G, 100 mW cm^–2^) at zero applied bias voltage to determine the long-term performance of the system. In contrast, the performance is mainly evaluated by tracking the amounts of products for standalone artificial leaves (where both electrodes are directly connected) and PC sheets.

Depending on the choice of substrates and reaction, a diverse range of products can be generated from PEC artificial leaves and PC sheets. Gaseous products such as H_2_, syngas, and O_2_ accumulate in the headspace of the reactor with time. These can be detected and quantified using gas chromatography either in-line or through the manual injection of a specific volume from the headspace. On the other hand, liquid products formed from the oxidation of waste substrates (e.g., formic acid, glycolic acid, and glyceric acid) or CO_2_ reduction (e.g., formate, acetate, and alcohols) can be analyzed using a combination of high-performance liquid chromatography, ion chromatography, and NMR spectroscopy.

## 4 Performance Metrics

Artificial photosynthesis systems are commonly evaluated by their solar-to-fuel conversion efficiency (η_STF_), which can be calculated using formula 1, where *r*_product_ is the product rate, *ΔG* is the reaction’s Gibbs free energy (*ΔG* > 0), *P* is the total incident solar power, and *A* is the irradiated area.^8^
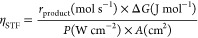
1

In the case of PEC systems, η_STF_ can be expressed as shown in formula 2, where *J* is the photocurrent density, FY is the Faradaic yield, and *ΔE* is the thermodynamic cell potential (*ΔE* < 0) between the two electrodes. This cell potential amounts to –1.23 V for water splitting and –1.33 V when coupling water oxidation to CO production at 298 K.^53^
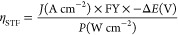
2

A comparison between solar fuel systems only by η_STF_ poses challenges when considering photocatalysis processes with a negative Gibbs free energy (*ΔG* < 0).^54^ As shown above, *ΔG* is mainly dependent on water oxidation, meaning that the η_STF_ value can be high for water and CO_2_ splitting (*ΔG* ≫ 0), whereas the η_STF_ of organic conversions will be per definition low (Δ*G* ≈ 0). However, some products are more valuable than others. Hence, as we expand the chemistry scope toward more complex oxidation reactions, the η_STF_ does not reflect the true economic value of a solar chemical process. To address this limitation, we propose a number of alternative metrics for solar fuel production, which take the economic value of products into account. These metrics focus on the areal performance instead of the solar energy conversion efficiency.

A first general metric is the amount of product (*n*_product_) synthesized per area (*A*) and time (*t*), which corresponds to the rate of conversion per area (*r*_product_).
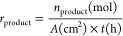
3

This can form the basis of other value-oriented metrics. A simplified solar-to-value (STV) creation rate can be obtained by subtracting the total value of substrates (∑_*j*=1_^*m*^*C*_*j*_ × *n*_*j*,substrate_) from the value of products (∑_i=1_^*n*^*C*_*i*_ × *n*_*i*,product_), as shown in eqs 4 and 5, where *C*_*i*_ is the cost of a chemical *i*. This STV metric can be further expanded to include the costs of substrate pretreatment, product separation and storage, other process costs, and the factor for scale, although calculating such values will require an extensive technoeconomic analysis. In the long term, such metrics may also consider the levelized cost of chemicals (analogous to the levelized cost of energy), measuring the average net value of chemicals for a solar plant over its lifetime.
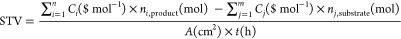
4
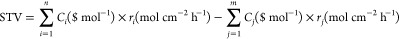
5

These metrics are particularly suitable for organic transformations, as the conversion rate is limited only by the photocurrent or light absorption of the semiconductor, which are determined by its external quantum efficiency (EQE) over the visible spectrum.^4^ Moreover, the value of waste and CO_2_ can be negative due to tipping fees and carbon taxes, respectively, meaning that value is created from both substrates (waste mitigation) and products (H_2_, CO, and organics). As the prices of chemicals fluctuate, value-driven metrics should be updated in dedicated databases analogous to traded commodity indices, while the areal conversion rates would constitute fixed reported values.

From this point of view, PEC reforming would stand out when compared to its PC analogues and would even approach the performance of state-of-the-art wired PV–EC systems for overall water splitting.^55^ In our case, the product rates of perovskite artificial leaves for waste PEC reforming were improved 5–10 times over that of the perovskite–BiVO_4_ devices for water splitting and >3000 times over the photocatalytic CN_*x*_ panels for PET reforming, as shown in Table 1.^4^

## 5 Reactor Engineering and Upscaling

### 5.1 Setup Types

**Figure 3:**
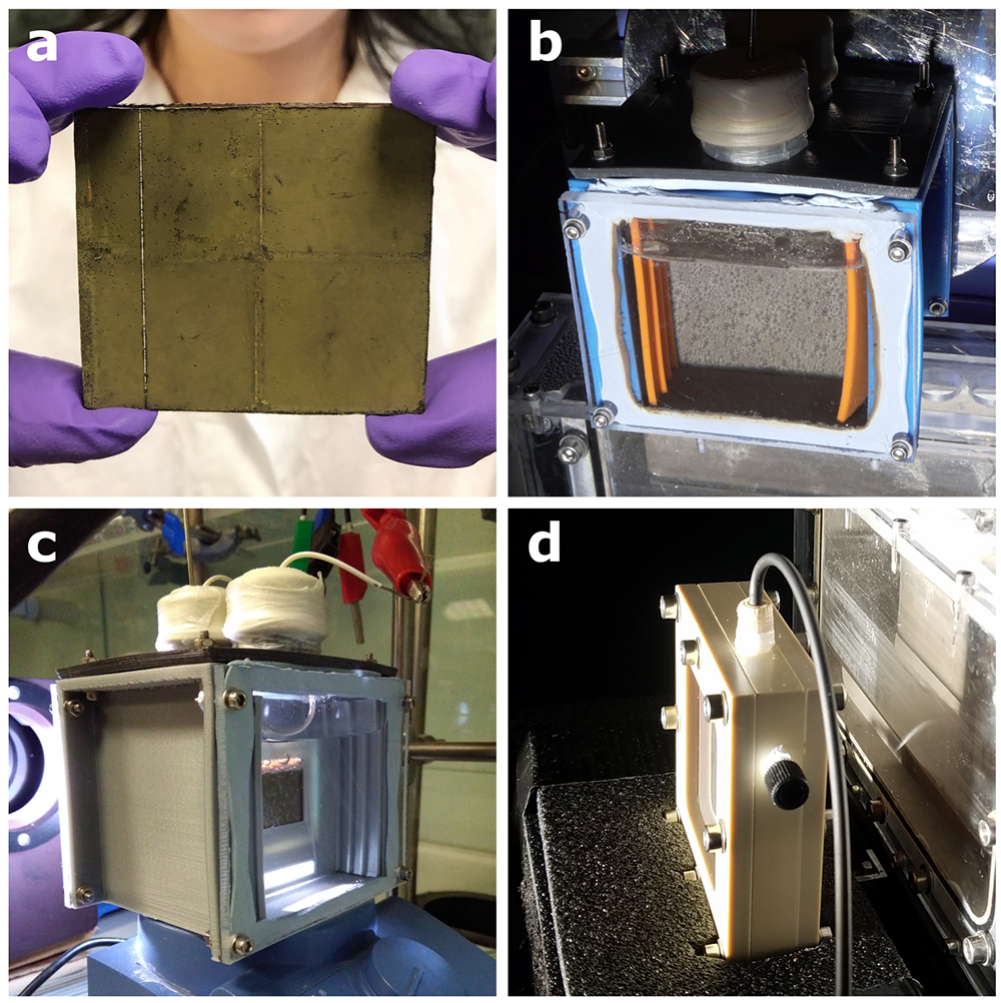
Upscaling. (a) Image of a 5 × 5 cm^2^ PC sheet. (b) Same PC sheet under operation in a 3D-printed reactor. (c) 2 × 2 cm^2^ PEC leaf in the 3D-printed reactor. (d) PC sheet in a machined flow reactor for photoreforming (5 × 5 cm^2^ window). Adapted with permission from ref (3). Copyright 2020 Wiley-VCH GmbH. (b, c) Batch reactors. (d) Flow reactor.

Most studies of PEC or PC systems are performed in glassware reactors, which restrict the sample size. In contrast, scalability studies require custom-made reactors, which can be fabricated through machining from a solid block of material,^3^ from transparent commercial panels,^57,58^ or by modern fabrication techniques such as 3D printing (Figure 3).^29,59^ Reactors machined from polyether ether ketone (PEEK) are more suitable for waste photoreforming studies, as PEEK can withstand the strongly basic solution (pH ≈ 14) often employed under operation.^3^ On the other hand, 3D printing offers the unique possibility of fast, inexpensive prototyping, as modular reactors can be printed from poly(lactic acid) (PLA) overnight and assembled the next day. These versatile 3D-printed reactors can be easily adapted to accommodate PEC leaves,^29^ PC sheets,^2^ or individual photoelectrodes of various sizes,^29,51^ in two- or three-electrode configurations. 3D printing holds particular promise as more resistant and versatile materials are constantly becoming available for printing.

Reactors can be further categorized into batch or flow systems. Similar considerations apply for batch and flow reactors using panels, as for those using photocatalyst suspensions.^60,61^ However, since the light absorber is immobilized, care must be taken in regard to the panel irradiation (direct or through liquid), solution turbidity, and reactor geometry. Batch reactors are completely closed; a set quantity of reactants and the resulting products are contained within a set volume for a given time frame. In flow reactors, the reactant(s) are circulated over an immobilized photocatalyst and the products are collected in a separate vessel. While batch reactors are typically easier to design and construct, they face practical challenges upon scaling, including mass transfer limitations due to stirring inconsistencies across the reactor area and overpressure development due to gaseous product generation. In contrast, flow reactors maintain constant mixing for improved mass transfer and can provide facile separation and in-line quantification of products and reactants.^62^ For slow reactions such as photoreforming, it is crucial to carefully adjust the flow rate to allow for maximal retention time while maintaining sufficient mass transfer.

Typically, oxidative and reductive gaseous products are obtained on different sides of the panel for artificial leaves, which facilitates their separation. In contrast, O_2_ and H_2_ are produced in close proximity during water splitting, on the same side of the PC sheets. Due to the wide flammability range of H_2_ (4–94% at ambient temperature and pressure), the safe separation of H_2_ from gaseous mixtures requires careful consideration. When a commercial polyimide membrane was used, 73% of H_2_ was safely separated and recycled from the moist gas mixture produced by overall water splitting over 100 m^2^ photocatalyst sheets.^38^ However, more than 20% of H_2_ remained in the feed gas. Gas separation membranes with a higher H_2_ permeability and lower O_2_ permeability will be required for operation in the future. These issues can be avoided through photoreforming, as the organic substrates can be selectively oxidized to aqueous products. Hence, clean H_2_ accumulates in the headspace, while oxidative products remain dissolved in solution.

In terms of irradiation, laboratory setups often employ a broadband Xe light source, which is fitted with an AM 1.5G filter to match the output light to the solar spectrum. UV filters (e.g., >420 nm) can be further attached to avoid the degradation of sensitive synthetic or biological components, whereas IR water filters mitigate reactor heating. Neutral density filters of varying opacity can also be used to simulate seasonal changes in sunlight intensity, regions with lower solar irradiance, or overcast weather conditions.^1^ However, calibrated commercial setups only provide irradiation sizes below 30 × 30 cm^2^, which limits the number and scope of scalability studies. In contrast, low-cost light-emitting diode (LED) light sources are well established in organic flow photochemistry and the pharmaceutical industry.^63,64^ Such LED arrays provide control over the excitation wavelength, require a low power input, and are suitable for indoor use, which made them recently attractive for organic transformations using PC suspensions.^64^ Outdoor evaluation under real sunlight will ultimately be required and field testing allows performance assessment of solar chemistry panels under different weather conditions that influence light exposure and temperature.

### 5.2 Practical Advantages

From a sustainability perspective, thin film technologies are a crucial step toward reducing the environmental impacts of light-to-chemical conversion. These panel designs offer several general advantages for solar fuel production. In comparison to PV–EC systems, artificial leaves require no additional wiring, electronics, or membranes, which decreases the overall complexity of the system.^12^ Such PEC or PC panels are often based on earth-abundant elements and operate in benign, (nearly) neutral pH solutions, which can further decrease their cost of operation.

The energy use and greenhouse gas emissions of the additional materials and processing steps required to fabricate a PC sheet or artificial leaf are approximately 0.234–1.788 MJ m^–2^_panel_ and 0.016–0.101 kg_CO_2__ m^–2^_panel_, respectively, depending on the annealing temperature. These values include the environmental impacts associated with glass substrate production (assumed to be 2 mm thick) and annealing of the PC sheet or artificial leaf at temperatures of 80–450 °C (assumed to use an electric furnace); binders such as Nafion were neglected as they comprise a minimal portion of the overall material mass. Given that separating a photocatalyst by centrifugation would use 0.072 MJ m^–2^_irr_ and emit 0.004 kg_CO_2__ m^–2^_irr_ (assuming a 1 cm reactor depth and two separation steps with energy requirements of 1 kWh m^–3^_solution_ each),^65^ the impacts from immobilization would be negated after 3 to 25 reuse cycles. Separation by vacuum filtration has higher requirements of 0.306 MJ m^–2^_irr_ and 0.017 kg_CO_2__ m^–2^_irr_ (assuming a filter area of 100 cm^2^ and energy use of 8.5 kWh m^–2^);^66^ therefore, a PC sheet or artificial leaf could be environmentally beneficial after as little as one reuse cycle.

Catalyst recyclability is a key benefit of immobilization, as thin films can be easily retrieved from the reaction medium compared to homogeneous PC suspensions. For example, the compact Cu_30_Pd_70_|perovskite|Pt PEC artificial leaves have shown promising reusability over four cycles of PET waste reforming.^4^ CN_*x*_|Ni_2_P sheets have been similarly shown to retain over 70% of their H_2_ evolution activity after four reuse cycles of photoreforming under mildly alkaline conditions (0.5 M KOH), with minimal co-catalyst (0.3% Ni) and photocatalyst (negligible for CN_*x*_) leaching.^3^ This is a marked improvement over a particulate CN_*x*_|Ni_2_P system, which lost half of its efficiency after a single reuse cycle.^21^ For photoreforming systems, in particular, immobilization also enables the use of turbid waste streams that would otherwise prevent light from reaching a PC suspension. In this case, the transparent glass substrate of PC sheets and wired PEC systems can directly face the light source, acting as a window for the reactor.^3,4^

Furthermore, a PC sheet reactor can be designed to easily track the sun so that direct radiation can be captured more effectively during the day.^67^ When compared to a fixed panel array, the baseline-levelized production cost of a sun-tracking panel reactor integrated with a solar concentrator can be significantly reduced.^68^ The resulting elevated temperatures caused by the photothermal effect are also known to improve catalysis.^69−71^ Given the similar construction and implementation requirements for thin film solar fuel technologies and photovoltaics, artificial leaves and PC sheets are expected to benefit from expertise in the solar industry, resulting in more facile upscaling than for particulate systems.

### 5.3 Remaining Challenges

Despite their advantages, solar fuel panels suffer from similar scalability challenges as other light-harvesting technologies,^72^ resulting in a nonlinear scaling of the performance with the photoactive area (Table 2). One known challenge for artificial leaves is the buildup of a pH difference between the anodic and cathodic sides, which leads to an increase in the overpotential during operation and may affect the catalysis. This effect is particularly detrimental for large-scale devices operating under neutral pH solutions without solution convection.^73^ This can be avoided by utilizing electrolyte solutions with either a very low or high pH (as in the case of PEC waste reforming). Neutral pH solutions can also be employed using controlled convection streams (i.e., solution recirculation), whereas carefully positioned separators can mitigate product crossover.^74,75^ Alternatively, product separation can be attained in microfluidic flow reactors, where closely positioned channels are separated by appropriate ion-exchange membranes.^76^ On the other hand, resistive losses can occur through a conductive electrode substrate. For example, FTO glass limits the photocurrent of both perovskite photocathodes^29^ and BiVO_4_ photoanodes^51^ to below 100 mA. This can be avoided through a monolithic design, where the charge flow occurs only perpendicular to the panel surface (cross-plane).^15^

**Table 2:** Comparison between the Advantages and Disadvantages of Solar Panel Technologies

	Water splitting and CO_2_ reduction		Photoreforming	
	PEC leaf	PC sheet	PEC leaf	PC sheet
overall benefits	compact, standalone, facile retrieval and reuse, cost, off-grid applications			
overall challenges	(catalyst) optimization, translation to real-world applications, product collection			
light absorbers	tandem		single	
fabrication	complex	moderate	complex	simple
areal activity	high	moderate	very high	low
scalability	moderate	high	moderate	high
selectivity	high	high	high	moderate
stability	moderate	high/co-catalyst leaching	moderate	high
product separation	red. and ox. separated	same side	red. and ox. separated	same side
impurities and turbidity	side reactions, optical losses	compatible	optical losses	compatible

In principle, both issues can be circumvented by employing the PC sheet design, as charges travel only a small distance between adjacent particles.^17^ Consequently, a comparable solar-to-formate conversion efficiency was observed even if the active area was increased 20 times.^2^ However, a performance decrease is still sometimes observed for these systems,^3,17^ which can be linked to manufacturing inhomogeneity and mass transport limitations on a large scale. A careful optimization of (photo)catalytic deposition procedures and reactor design will therefore be necessary to minimize material inconsistencies, promote mass transport, and balance trade-offs between light absorption and charge recombination.

Besides fabrication, product separation, and scalability, current panel technologies can suffer from light absorber or catalyst degradation, which manifests through moisture infiltration or catalyst leaching. This limits the stability of state-of-the-art systems to several weeks, whereas oxide-based compounds have been shown to operate for several months under real-world conditions.^38^ Despite the interplay between stability and performance, we recently demonstrated that artificial leaves can also perform overall water splitting over several hundred hours by employing an oxide-based device structure and suitable hydrophobic encapsulation.^77^

Recent technoeconomic analysis has also suggested that PC sheets could be slightly more expensive than particulate systems (by ∼12%) due to increased reactor complexity and corresponding cost.^78^ However, these results are dependent on the assumed PC recyclability in the different systems. Our own technoeconomic analysis of (slurry) waste photoreforming showed that higher photocatalyst lifetimes of 1–10 years, which may be more feasible with a robust thin film system, can significantly reduce both cost and environmental impacts.^40^

## 6 Broader Reaction Scope and Applications

The solar chemistry panel technologies discussed in this Account can in principle be utilized for applications beyond solar fuels. Photoreforming with PEC leaf or PC sheet systems already addresses the waste management sector, as it is capable of converting a diverse range of biomass, plastics, and industrial by-products into organic chemicals. This reaction scope could be further expanded to other problematic waste streams containing pollutants,^79^ agricultural or medical waste, and especially opaque materials that prevent effective photoreforming under slurry conditions. It should nevertheless be noted that chemical, thermal, or biological pretreatments will likely be required to release soluble substrates that can easily diffuse to the thin film surface and undergo oxidation.

Moreover, both PC and PEC approaches may be employed in the synthesis of higher-value chemicals. While organic transformations are often performed in PC flow systems,^63^ the potential of photoelectrodes (for instance, Fe_2_O_3_) in synthesis has been recognized only recently.^80^ PEC systems in particular can offer controlled oxidation through the use of certain applied potentials or selective co-catalysts. This capability not only ensures a selective product stream that can have commercial value but also prevents the overoxidation of substrates to undesirable CO_2_.^4^ We have already demonstrated the production of value-added organic products (glycolic acid, glyceric acid, and formic acid, among others).^4^ The synthesis of cyclobutanes, which are building blocks for some pharmaceutical compounds, over immobilized CN_*x*_ has also been recently reported.^81^ With further advances in catalyst design, integrated panels could be applied to the production of fine chemicals or pharmaceuticals, helping to decarbonize industries that are currently reliant on fossil fuels for both feedstocks and reaction conditions (i.e., heat and pressure).

## 7 Conclusions and Outlook

Despite their differences in nomenclature, assembly, and operating principles, thin film solar panel technologies share a common design, which makes them stand out among light-harvesting technologies. Such panels have recently demonstrated their versatility for a wide range of reactions beyond water splitting, including CO_2_ reduction and organic transformations. For the latter, the lower thermodynamic threshold required to oxidize organics provides an advantage, which can enable those systems to match the production rates of conventional PV–EC systems. The integration of all components in standalone PEC artificial leaves results in a material cost reduction. Furthermore, the recyclability of such panels can offset the cost and environmental impacts associated with PC particle immobilization.

While these systems are yet mostly developed on a laboratory scale, further improvements in terms of scalability, stability, and performance could make them attractive from an economic perspective. Here, the modularity of the systems can provide a key advantage, as solar fuels panels would benefit from progress in the established electrocatalysis and photovoltaics communities.^77^ Once such systems can sustain competitive product rates over several years on a square-meter scale, they could be deployed for commercial applications. In a future scenario, light-driven panels may find use in fine chemical synthesis, where the value of the products outweighs the lower product rates compared to those of water and CO_2_ electrolysis. In the long term, such panels would ideally contribute to a circular fuel and chemicals economy, thereby closing the carbon cycle.
